# Serum Sickness following Tetanus Toxoid Injection

**DOI:** 10.1155/2021/6680979

**Published:** 2021-01-20

**Authors:** Saja alhawal, Manar Aldarwish, Zainab Almoosa

**Affiliations:** ^1^Department of Paediatrics, King Abdulaziz Hospital for National Guard, Alahsa, Saudi Arabia; ^2^Department of Paediatrics Infectious Diseases, Almoosa Specialist Hospital, Alahsa, Saudi Arabia

## Abstract

Serum sickness is an allergic reaction that frequently occurs in patients after the injection of foreign protein or serum. It is characterized by fever, skin rash, enlarged lymph nodes, and painful joints. In this case, we describe a case of 6-year-old girl who developed a rash and arthralgia after being vaccinated with tetanus toxoid injection after a cut wound.

## 1. Introduction

Serum sickness is a type III immune complex-mediated hypersensitivity reaction caused by exposure to an antigen and nonhuman serum proteins such as microbial antitoxins and venom antitoxins, vaccines, and immune and cell metabolism modulators, leading to the formation of antibodies against these antigens. The binding of these antigens to the specific antibodies will form immune complexes that get deposited in the tissues leading to inflammatory response through activation of the complement cascade [1–4].

It was first described in 1905 by two pediatricians von Pirquet and Schick when they introduced a horse serum-derived antitoxin against diphtheria and scarlet fever. They observed a reaction to the antitoxin in the form of fever, skin eruption, joint involvement, and lymphadenopathy [5, 6].

The symptoms triad of serum sickness consists of fever, rash, and joint involvement (arthralgia and arthritis), usually occurring 7–14 days after exposure to the offending agent. Other less common symptoms include lymphadenopathy, nonspecific headache, gastrointestinal complaints, blurred vision, and myalgia [2, 7].

Regarding prognosis, it is considered a self-limiting disease with a good prognosis usually resolving within a few weeks. The most vital aspect of management is the withdrawal of the causative agent. However, symptomatic treatment with antihistamines and NSAIDs can help [8–11].

### 1.1. Case Report

A previously healthy six-year-old Saudi girl presented with a one-day history of bilateral lower limb pain. The pain was extending from knees down to feet, severe enough to disable her from walking or even standing. Two days before this presentation, she had a cut wound in her right hand and was taken to the hospital where a Tetanus Toxoid injection was given (deltoid muscle). She denied any history of fever, trauma, or recent infection. The patient's vaccination history is up to date as per national guidelines; last routine vaccination for our case was 6 months prior to presentation (preschool vaccination). Saudi national vaccination schedule includes total of five doses of tetanus vaccine, three of them in the first year of life, the fourth is in the second year, and the fifth is at preschool age.

Upon examination, she was found to have swelling and tenderness of right foot and both elbows, without erythema or warmth. There was no palpable lymphadenopathy or hepatospleenomegaly.

Investigation showed a normal CBC (WBC 9,000 with normal differential, Hgb 12, and platelets 260), normal inflammatory markers (CRP 5 and ESR 8), and normal complement level (C3 90 mg/dL and C4 of 28 mg/dL).

Ultrasound showed mild oedematous changes in subcutaneous soft tissues along the dorsal aspect on the right foot. An impression of serum sickness was proposed and was managed with antihistamines and analgesics.

On the second day of admission, she developed a petechial nonpruritic rash involving both lower limbs that resolved spontaneously. There was no mucus membrane involvement. She showed significant improvement in her condition; by the third day, she could walk free of pain and no more joint swelling or skin rash.

Other differential diagnoses were entertained, like poststreptococcal arthritis, rheumatic fever, and brucellosis. These diagnoses were ruled out by history and clinical exam as well as laboratory and imaging modalities. Echocardiography (ECG) was normal, ASO titre was less than 200, and throat culture and rapid strep antigen test were both negative. Brucella serum agglutination test was normal (1 : 40).

The patient was seen as an outpatient one month after discharge. She was fine and completely asymptomatic. We emphasized taking the Tdap booster dose at the age of 11 years with careful monitoring as similar reaction may occur.

## 2. Discussion

Tetanus is a serious complication of wounds and injuries. Prophylaxis in routine wound management is a major strategy for tetanus prevention in the Emergency Department.

However, individuals who receive the toxoid may have adverse events, including injection site reaction (local pain, erythema), fever, nausea, and arthralgia.

Severe anaphylactic reactions, GuillainBarré syndrome (GBS), and brachial neuritis attributable to tetanus toxoid, though rare, have been reported as well.

Serum sickness (SS) is an immune complex-mediated hypersensitivity reaction (Type III), which usually begins 7 to 10 days (occasionally as late as three weeks) after the administration of drugs, foreign proteins, or infections. About 90% of cases will manifest classically with fever, polyarticular arthralgia, lymphadenopathy, and cutaneous symptoms [12, 13].

Furthermore, a similar entity called serum sickness-like reaction (SSLR) clinically resembles serum sickness. The difference relies on that SSLR is usually less severe with low-grade fever or no fever at all. The other difference is that the causing agents (e.g., antibiotics, infections, and antiepileptic medications), and the underlying pathogenesis is not fully understood as serum sickness. However, it is managed the same way as serum sickness [14, 15].

Although SS reactions are rare secondary to vaccines, we found several reported cases of SS induced by rabies, rubella, inactivated influenza, pneumococcal, and hepatitis B vaccines [14–18].

In terms of reported cases on vaccines causing SS, there was one case report of serum sickness after Tetanus Immunization in June 1972 in a seven-year-old boy who developed typical serum sickness about three days after injection of diphtheria and tetanus toxoids [19].

In our case, we described the events of rash and joint involvement in six-year-old Saudi girl three days following a Tetanus Toxoid injection, which we believed to be SS or SSLR secondary to tetanus toxoid.

In moderate or severe SS cases, laboratory findings can show leucocytosis and thrombocytopenia, elevated ESR and C-reactive protein, mild proteinuria, or haematuria. Decreased complement levels, including C3, C4, and total hemolytic complement (CH50), reflect complement consumption.

However, our patient's workup was within normal, which can be found in most patients with mild SS.

Some might argue that our case's presentation is more consistent with serum sickness-like reaction rather than serum sickness because of mild presentation, no fever, and normal inflammatory markers. Furthermore, the presentation timeline is not typical for SS, where symptoms typically start seven to ten days after the causative agent. In comparison, in our case, the first symptom started three days after the Tetanus Toxoid. However, this atypical timeline can be unique to Tetanus Toxoid.

It is usually difficult to definitively determine that the tetanus toxoid was the leading cause to explain the patient's symptoms but is likely to be blamed because of the events' temporal relationship.

There is no definitive diagnostic test for serum sickness. Laboratory studies do not help to establish a diagnosis of SS or SSLR.

Serum sickness is self-limited and usually resolves within 1 to 2 weeks; therefore, treatment is symptomatic relief. Antihistamines may be administered to relieve pruritus. Nonsteroidal anti-inflammatory drugs are given for fever and joint pain, and, if necessary, steroids may be needed, especially in severe cases [14].

The prognosis is excellent in most serum sickness cases, with complete resolution of signs and symptoms in a few days like in our case.

## Data Availability

The data used to support the findings of this study are available from the corresponding author upon request.

## References

[1] Lawley T. J., Bielory L., Gascon P., Yancey K. B., Young N. S., Frank M. M. (1984). A prospective clinical and immunologic analysis of patients with serum sickness. *New England Journal of Medicine*.

[2] Frank M. M., Hester C. G., Adkinson N. F., Bochner B. S., Burks W. (2014). Immune complex–mediated diseases. *Middleton’s Allergy Principles and Practice*.

[3] Ryan N. M., Kearney R. T., Brown S. G. A., Isbister G. K. (2016). Incidence of serum sickness after the administration of Australian snake antivenom (ASP-22). *Clinical Toxicology*.

[4] Sicherer S. H., Leung D. Y. M., Kliegman Serum sickness. *Nelson Textbook of Pediatrics*.

[5] Shulman S. T. (2017). Clemens von Pirquet: a remarkable life and career. *Journal of the Pediatric Infectious Diseases Society*.

[6] von Pirquet C. F., Schick B., Schick B. (1905). *Serum Sickness*.

[7] Bielory L., Gascon P., Lawley T. J., Young N. S., Frank M. M. (1988). Human serum sickness. *Medicine*.

[8] Erffmeyer J. E. (1986). Serum sickness. *Annals of Allergy*.

[9] Clark B. M., Kotti G. H., Shah A. D., Conger N. G. (2006). Severe serum sickness reaction to oral and intramuscular penicillin. *Pharmacotherapy*.

[10] Tatum A. J., Ditto A. M., Patterson R. (2001). Severe serum sickness-like reaction to oral penicillin drugs: three case reports. *Annals of Allergy, Asthma & Immunology*.

[11] Chiong F. J., Loewenthal M., Boyle M., Attia J. (2015). Serum sickness-like reaction after influenza vaccination. *BMJ Case Reports*.

[12] Marcdante K. J., Kliegman R. M., Kliegman D. W., Brady M. T., Jackson M. A., Long S. S. (1985). Serum sickness. *Nelson Essentials of Pediatrics*.

[13] http://ebooks.aappublications.org/content/red-book-30th-edition-2015

[14] Apisarnthanarak A., Uyeki T. M., Miller E. R., Mundy L. M. (2009). Serum sickness-like reaction associated with inactivated influenza vaccination among Thai health care personnel: risk factors and outcomes. *Clinical Infectious Diseases*.

[15] Arkachaisri T. (2002). Serum sickness and hepatitis B vaccine including review of the literature. *Journal of the Medical Association of Thailand*.

[16] Warrington R., Martens C., Rubin M., Rutherford W., Aoki F. (1987). Immunologic studies in subjects with a serum sickness-like illness after immunization with human diploid cell rabies vaccine. *Journal of Allergy and Clinical Immunology*.

[17] George R., Thompson M. D., Alfredo Ferreyra M. D., Robert G. (1971). Acute arthritis complicating rubella vaccination. *Arthritis & Rheumatism*.

[18] Hengge U. R., Scharf R. E., Kroon F. P., Pfeffer K. (2006). Severe serum sickness following pneumococcal vaccination in an AIDS patient. *International Journal of STD & AIDS*.

[19] Daschbach R. J. (1972). Serum sickness and tetanus immunization. *JAMA: The Journal of the American Medical Association*.

